# Development and Internal Validation of the Yuvarajan Sarcoidosis Diagnostic Score (YSDS): A Retrospective Cohort Study

**DOI:** 10.7759/cureus.89046

**Published:** 2025-07-30

**Authors:** Yuvarajan S, Praveen Radhakrishnan, Durga Krishnamurthy, Navya Cherukkumalli, Sagana Ravikumar

**Affiliations:** 1 Department of Respiratory Medicine, Sri Manakula Vinayagar Medical College and Hospital, Puducherry, IND; 2 Department of Obstetrics and Gynaecology, Sri Lakshminarayana Institute of Medical Sciences, Puducherry, IND

**Keywords:** hilar adenopathy, non-caseating granuloma, novel sarcoidosis score, pulmonary sarcoidosis, yuvarajan sarcoidosis diagnostic score

## Abstract

Background: Sarcoidosis is a complex, multisystem granulomatous disease of unknown etiology, often presenting a diagnostic challenge due to its highly variable clinical manifestations and its overlap with infectious and neoplastic diseases. This is especially problematic in regions with a high burden of tuberculosis (TB), such as India, where the clinical and radiological features of sarcoidosis and TB can be remarkably similar. Early, accurate diagnosis is imperative to guide treatment and avoid inappropriate therapy, yet no universally accepted diagnostic scoring system exists.

Objective: The objective of this study was to develop and internally validate a novel, composite clinical scoring tool named the Yuvarajan Sarcoidosis Diagnostic Score (YSDS) to aid in the diagnosis of sarcoidosis using routinely available clinical, radiologic, laboratory, and histopathologic parameters.

Methods: A retrospective observational study was conducted at a tertiary care hospital in South India. Medical records of 94 patients evaluated for suspected sarcoidosis between January 2022 and January 2025 were reviewed. Patients were categorized into sarcoidosis (n = 63) and non-sarcoidosis groups (n = 31) based on histopathological confirmation, radiological features, and exclusion of differential diagnoses. Multivariate logistic regression was used to identify significant independent predictors of sarcoidosis. These predictors were used to create a weighted diagnostic score, and their diagnostic accuracy was assessed using receiver operating characteristic (ROC) curve analysis.

Results: Five independent predictors were identified: bilateral hilar lymphadenopathy (BHL) on chest imaging, elevated serum angiotensin-converting enzyme (ACE) levels, histologic presence of non-caseating granulomas, negative Mantoux test, and characteristic extrapulmonary manifestations such as uveitis, parotid gland enlargement, or lupus pernio. Each parameter was assigned a score based on the regression coefficient. The YSDS score ranged from 0 to 13, with a cutoff ≥8 yielding a sensitivity of 87.3% (55/63), specificity of 83.9% (26/31), positive predictive value (PPV) of 89.6% (55/61), negative predictive value (NPV) of 80.6% (26/33), and an overall accuracy of 85.9% (81/94). The area under the ROC curve was 0.90, indicating excellent discriminatory power.

Conclusion: The YSDS is a statistically robust, easy-to-implement clinical tool that enhances diagnostic confidence in sarcoidosis, particularly in settings where TB and other granulomatous diseases are prevalent. It offers a promising strategy for standardized diagnostic assessment and warrants external validation in larger, prospective cohorts.

## Introduction

Sarcoidosis is a multisystem granulomatous disease for which the aetiology is not completely understood but is believed to result from a combination of genetic susceptibility and exposure to specific environmental or infectious triggers. Histologically, it is characterized by the presence of non-caseating granulomas in affected tissues. Although the disease has been recognized for over a century, it continues to pose significant diagnostic challenges due to its protean clinical manifestations and overlap with other granulomatous disorders. While the lungs and intrathoracic lymph nodes are most commonly involved, sarcoidosis can affect virtually any organ system, adding to its broad clinical spectrum and diagnostic complexity [1].

Globally, the burden of sarcoidosis varies significantly by geography, ethnicity, and socio-environmental conditions. In Western countries, especially among Scandinavian and African-American populations, sarcoidosis is more frequently diagnosed and extensively studied [2]. The estimated annual incidence in the United States ranges from five to 40 cases per 100,000 population, with higher rates reported among African-Americans [2]. In contrast, in regions such as India and sub-Saharan Africa, where tuberculosis (TB) is endemic, sarcoidosis remains underrecognized and frequently misdiagnosed [3]. While exact national prevalence data for sarcoidosis in India are limited due to underreporting and diagnostic challenges, smaller regional studies suggest an estimated incidence of 10 to 12 cases per 100,000 population [3]. However, this figure is likely an underestimation, as many cases are either missed or misattributed to TB due to significant clinical and radiologic overlap between the two conditions. Both diseases can present with fever, cough, bilateral hilar lymphadenopathy (BHL), pulmonary infiltrates, elevated inflammatory markers, and granulomatous inflammation on histopathology [3]. Such resemblance can result in diagnostic confusion and inappropriate administration of anti-tubercular therapy (ATT), which may delay the correct diagnosis and lead to suboptimal patient outcomes [3].

Currently, the diagnosis of sarcoidosis remains fundamentally a diagnosis of exclusion. It necessitates a comprehensive and integrative assessment that combines clinical evaluation, characteristic radiologic patterns, supportive laboratory findings, histopathologic demonstration of non-caseating granulomas, and a systematic exclusion of alternative causes of granulomatous inflammation, particularly TB in endemic regions [1,4,5]. Laboratory investigations such as elevated serum angiotensin-converting enzyme (ACE), hypercalcemia, and hypercalciuria may provide supportive evidence, though they lack specificity. Several international guidelines, including those developed by the American Thoracic Society (ATS), the European Respiratory Society (ERS), and the World Association for Sarcoidosis and Other Granulomatous Disorders (WASOG), offer structured diagnostic frameworks to aid this integrative process [1]. However, these recommendations are largely consensus-driven, qualitative in nature, and often dependent on expert interpretation. Crucially, they do not provide standardized, quantifiable scoring tools that can facilitate diagnostic decision-making at the point of care, particularly in resource-limited settings where access to advanced diagnostics and multidisciplinary expertise may be constrained [6].

This diagnostic subjectivity is particularly problematic in TB-endemic countries like India, where distinguishing sarcoidosis from TB is a routine clinical challenge. In such contexts, clinicians are often compelled to rely on empirical judgment or therapeutic trials of ATT in unclear cases. This approach not only delays the initiation of appropriate therapy for sarcoidosis but may also expose patients to unnecessary treatment-related toxicity. A validated diagnostic scoring system that integrates common clinical, radiologic, laboratory, and histopathologic features would offer a structured, evidence-based aid to improve diagnostic accuracy and consistency in such settings.

To date, very few diagnostic tools have been designed specifically for use in TB-endemic regions. India, with its dual burden of TB and increasing recognition of sarcoidosis, represents a crucial setting for the development of such a tool [3,7]. In response to this clinical need, we developed the Yuvarajan Sarcoidosis Diagnostic Score (YSDS), a pragmatic, statistically derived scoring system that combines routinely available clinical and investigative data to assist clinicians in differentiating sarcoidosis from its mimics.

The objective of this study is to develop a clinically implementable composite diagnostic score based on independent predictors of sarcoidosis and to internally validate this score using robust statistical methods, thereby establishing its reliability and utility in real-world clinical practice.

## Materials and methods

Study design and setting

This retrospective observational cohort study was conducted at the Department of Respiratory Medicine, Sri Manakula Vinayagar Medical College and Hospital (SMVMCH), Puducherry, India. SMVMCH is a tertiary care academic hospital serving a large referral population across South India and follows a structured interdepartmental diagnostic protocol for evaluating suspected granulomatous lung diseases.

The study adhered to the principles outlined in the Declaration of Helsinki and complied with institutional ethical guidelines. Owing to the retrospective nature of the study and the use of anonymised clinical data, a waiver of informed consent and institutional review board (IRB) approval was obtained. To ensure patient confidentiality, all medical records were de-identified prior to data extraction.

Study duration and patient selection

The study was conducted over a three-year period from January 2022 to January 2025. All patients aged 18 years or older who underwent evaluation for suspected sarcoidosis in the Department of Respiratory Medicine during this time were considered for inclusion.

Patients were eligible for inclusion if there was a documented clinical suspicion of sarcoidosis, which was standardized as the presence of unexplained respiratory symptoms (such as persistent cough, dyspnea, or chest discomfort) in combination with abnormal chest imaging findings suggestive of sarcoidosis, such as BHL or upper lobe-predominant infiltrates, reticulonodular opacities on chest radiograph, or high-resolution computed tomography (HRCT). Inclusion also required the availability of chest imaging (either a chest radiograph or HRCT), documented results of serum ACE levels, and tuberculin skin testing (Mantoux test). Furthermore, histopathological examination reports from relevant tissue samples, such as lymph node, lung, skin, salivary gland, or transbronchial biopsy, demonstrating granulomatous inflammation, were mandatory. Only patients with complete clinical records, including demographic details, presenting symptoms, and documented assessment of extrapulmonary organ involvement, were included in the final analysis.

Patients were excluded if they had a known diagnosis of HIV infection or were in an immunocompromised state, such as solid organ transplant recipients or individuals receiving long-term immunosuppressive therapy (e.g., for autoimmune diseases or post-transplant care). Patients on short-term corticosteroid therapy for other indications were not excluded, provided they did not meet other exclusion criteria. The rationale for excluding immunocompromised individuals was to avoid confounding factors that could alter the clinical presentation, radiologic features, or immune response relevant to sarcoidosis. Patients with incomplete diagnostic workups or missing key data elements such as chest imaging, Mantoux test results, ACE levels, or histopathologic confirmation were also excluded due to insufficient information for accurate classification. Additional exclusion criteria included coexisting microbiologically or histopathologically confirmed active TB at the time of presentation, known malignancies (including lymphoma or metastatic carcinoma), pregnancy, and age under 18 years. These exclusions were applied to minimize diagnostic ambiguity and ensure a homogeneous cohort for analysis.

Data collection and variables

Data were collected from the hospital’s electronic medical record system and cross-referenced with physical case records when necessary. Demographic data such as age, sex, smoking status, and presence of comorbidities were recorded. Clinical presentation was assessed with particular attention to respiratory symptoms, including dry cough, dyspnea, and chest pain, as well as extrapulmonary manifestations like visual disturbances, facial swelling, and cutaneous lesions.

Radiological data included findings from chest radiographs and HRCT scans, with emphasis on identifying typical features of sarcoidosis, such as BHL, pulmonary nodules, parenchymal infiltrates, and fibrotic changes. Laboratory investigations included serum ACE levels, which were considered elevated if greater than 52 U/L, and Mantoux test results, interpreted as positive when the induration was ≥10 mm and negative when <5 mm. Histopathological findings from biopsy samples were analysed for evidence of granulomatous inflammation, necrosis, lymphocytic infiltration, or malignancy. The presence of extrapulmonary organ involvement was documented based on clinical and radiological assessments, such as uveitis confirmed by ophthalmologic evaluation, parotid gland enlargement, or characteristic cutaneous lesions like lupus pernio.

Patient classification

Patients were classified into two groups based on clinical, radiological, histological, and microbiological findings. The sarcoidosis group comprised individuals who satisfied the diagnostic triad of non-caseating granulomas on biopsy, compatible clinical and/or radiological features, and exclusion of infectious causes, especially TB, as well as malignancy. The non-sarcoidosis group included patients who were initially suspected to have sarcoidosis but were ultimately diagnosed with other conditions such as TB, hypersensitivity pneumonitis, or lymphoma, based on histological confirmation, microbiological testing, and clinical course.

Score development and statistical analysis

All statistical analyses were performed using IBM SPSS Statistics for Windows, version 25.0 (IBM Corp., Armonk, NY, USA). Descriptive statistics were used to summarize baseline patient characteristics. Continuous variables were reported as means with standard deviations (SD) and were compared using either the independent t-test or the Mann-Whitney U test, depending on the distribution of the data. Categorical variables were presented as frequencies and percentages, with comparisons made using the chi-square test or Fisher’s exact test, as appropriate.

To identify independent predictors of a confirmed diagnosis of sarcoidosis, multivariate logistic regression analysis was employed. Variables with a p-value less than 0.10 in univariate analysis were entered into the multivariate model. Adjusted odds ratios (aOR), along with 95% confidence intervals (CI) and corresponding p-values, were calculated and reported.

A diagnostic scoring system, the YSDS, was developed using the beta (β)-coefficients from the final multivariate regression model. Each predictor was assigned a weighted score proportional to its β-coefficient, and the cumulative score was calculated for each patient. The optimal diagnostic cutoff value for the score was determined using Youden’s Index.

The diagnostic performance of the YSDS was evaluated using receiver operating characteristic (ROC) curve analysis. The area under the curve (AUC) was calculated along with sensitivity, specificity, positive predictive value (PPV), negative predictive value (NPV), and overall diagnostic accuracy. To ensure internal validity and reduce the risk of overfitting, bootstrapping was performed with 1,000 resamples. All statistical tests were two-tailed, and a p-value of less than 0.05 was considered statistically significant.

## Results

Demographics and clinical characteristics

A total of 94 patients were included in the study, of whom 63 (67%) were diagnosed with sarcoidosis and 31 (33%) with other confirmed diagnoses. The mean age of the cohort was 41.3 ± 9.5 years, with a slight female predominance (n = 53, 56.4%). Among the patients diagnosed with sarcoidosis, 54 (85.7%) presented with respiratory symptoms such as cough and dyspnea, while 15 (23.8%) exhibited extrapulmonary manifestations including uveitis and parotid gland enlargement. In the non-sarcoidosis group, the final diagnoses included TB in 20 patients, lymphoma in eight patients, and hypersensitivity pneumonitis in three patients. Multivariate logistic regression identified five independent predictors significantly associated with sarcoidosis (Table 1).

**Table 1:** Predictors of sarcoidosis OR: odds ratio; CI: confidence interval; ACE: angiotensin-converting enzyme; YSDS: Yuvarajan Sarcoidosis Diagnostic Score

Variable	Odds ratio (95% CI)	p-value	Weight in YSDS
Bilateral hilar lymphadenopathy	5.6 (2.4–13.1)	<0.001	3
Elevated serum angiotensin-converting enzyme (>52 U/L)	3.8 (1.5–9.2)	0.004	2
Non-caseating granulomas on biopsy	7.4 (3.2–17.3)	<0.001	4
Negative Mantoux test (<5 mm induration)	2.9 (1.2–7.0)	0.018	2
Extrapulmonary features (e.g., uveitis)	2.7 (1.1–6.5)	0.025	2

Diagnostic score calculation

**Figure 1:**
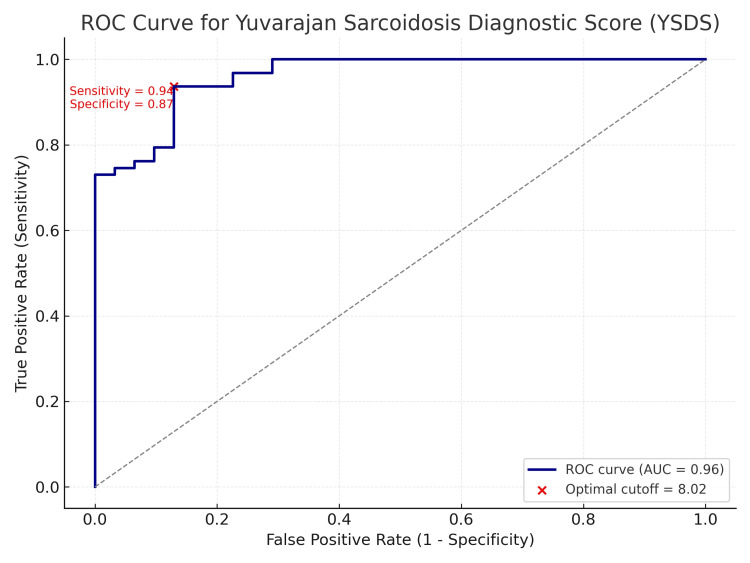
Receiver operating characteristic (ROC) curve for the Yuvarajan Sarcoidosis Diagnostic Score (YSDS). The area under the curve (AUC) is 0.90, indicating excellent diagnostic discrimination. The optimal cutoff score (≥8), identified using the Youden Index, yields a sensitivity of 87.3% and specificity of 83.9%.

The total score ranged from 0 to 13. An optimal cutoff of ≥8 was chosen based on maximum diagnostic performance (Table 2, Figure 1).

**Table 2:** Diagnostic performance of the Yuvarajan Sarcoidosis Diagnostic Score (YSDS)

Metric	Result
Sensitivity	87.3% (55/63)
Specificity	83.9% (26/31)
Positive predictive value (PPV)	89.6% (55/61)
Negative predictive value (NPV)	80.6% (26/33)
Accuracy	85.9% (81/94)
Area under the ROC curve (AUC)	0.90

These results suggest that the YSDS is a reliable and accurate tool for diagnosing sarcoidosis.

## Discussion

To the best of our knowledge, the YSDS is the first diagnostic scoring system for sarcoidosis developed in India, derived using real-world, clinically relevant data and robust statistical modelling. It integrates both classical features and under-recognized diagnostic clues characteristic of sarcoidosis. Each component of the score, BHL, elevated serum ACE, non-caseating granulomas on histology, a negative Mantoux test, and evidence of extrapulmonary involvement, has individual diagnostic value. When combined, these elements demonstrate excellent discriminatory ability in differentiating sarcoidosis from other granulomatous diseases [8-11].

Although the Sarcoidosis Diagnostic Score (SDS) [12] has previously been validated across multicontinental cohorts, including centers in India, it was not developed in an Indian context. A comprehensive literature review did not identify any prior Indian-origin diagnostic scoring tools specifically designed for sarcoidosis, highlighting the novelty and potential clinical relevance of the YSDS in TB-endemic settings.

Our findings are congruent with existing literature on the diagnostic workup of sarcoidosis. BHL remains a hallmark radiologic feature, particularly in Stage I sarcoidosis, where it appears without accompanying pulmonary infiltrates. Its diagnostic utility is well established, especially when the lymphadenopathy is symmetrical and non-infectious  [13-15]. Sarcoidosis is classically staged radiographically (based on chest radiograph findings) into four stages: Stage I (BHL alone), Stage II (BHL with pulmonary infiltrates), Stage III (pulmonary infiltrates without BHL), and Stage IV (fibrotic changes including volume loss and architectural distortion). This staging helps to assess disease extent and predict clinical course, though it does not always correlate directly with symptom severity. Serum ACE, although non-specific, reflects granuloma burden and is elevated in approximately 60% to 80% of active sarcoidosis cases [16]. However, ACE levels should be interpreted cautiously, as they may also be elevated in other systemic conditions such as diabetes mellitus, hyperthyroidism, and liver disease [17].

Histologic demonstration of non-caseating granulomas is the gold standard for sarcoidosis diagnosis, but granulomas can also occur in infections, malignancy, berylliosis, and autoimmune disorders [18-20]. Thus, a multiparametric tool like the YSDS, which contextualizes histologic findings with systemic features, offers more reliable diagnostic certainty.

The inclusion of extrapulmonary features such as uveitis, lupus pernio, or parotid gland enlargement is a unique strength of this scoring system. These manifestations, although infrequent, are highly specific for sarcoidosis and are typically underutilized in routine diagnostic algorithms [21-23]. For example, Heerfordt’s syndrome (uveoparotid fever), though rare, is virtually diagnostic of sarcoidosis when present [23].

A negative Mantoux test (<5 mm) was another independent predictor in our model. Tuberculin anergy in sarcoidosis results from impaired delayed-type hypersensitivity and dysfunctional regulatory T-cell responses [3,24]. In a TB-endemic country like India, the discriminatory value of a negative Mantoux test becomes particularly useful when applied in combination with other findings.

This diagnostic model is designed to be resource-efficient. Unlike diagnostic modalities such as fluorine-18 fluorodeoxyglucose positron emission tomography/computed tomography (18F-FDG PET/CT), which are expensive and not readily accessible in many Indian healthcare settings, all components of the YSDS rely on investigations commonly available even in secondary-level hospitals [16,25]. Hence, the YSDS has high applicability across varying levels of healthcare infrastructure.

From a global perspective, sarcoidosis is a clinical mimicker with a broad differential diagnosis, often overlapping with TB, fungal infections, hypersensitivity pneumonitis, and lymphoma. In regions where TB is endemic, empirical ATT is frequently initiated in uncertain cases, sometimes leading to misdiagnosis, treatment delays, or inappropriate deferral of corticosteroids [14,15]. A structured diagnostic tool like the YSDS may help mitigate such risks by providing a standardized, evidence-based assessment algorithm that reduces diagnostic uncertainty and prevents anchoring bias.

Existing frameworks, such as the A Case Control Etiologic Study of Sarcoidosis (ACCESS) criteria and the World Association of Sarcoidosis and Other Granulomatous Disorders (WASOG) organ assessment instrument, primarily emphasize clinical patterns and organ involvement but do not provide a quantitative scoring mechanism for point-of-care decision-making  [5,6,26]. Unlike ACCESS, which functions as a classification tool for research purposes, and WASOG, which relies on levels of diagnostic confidence without assigning scores, the YSDS introduces a quantifiable, user-friendly scoring system that synthesizes radiologic, laboratory, and histopathologic features into a cohesive diagnostic approach. This makes YSDS particularly valuable in resource-limited or high TB-burden settings. Furthermore, it lays the groundwork for future integration into electronic medical records, mobile diagnostic applications, or AI-assisted clinical decision support systems [24,26].

Importantly, the YSDS has the potential to evolve beyond diagnosis. In future studies, it could be used to stratify patients by disease severity or to predict therapeutic response. Biomarkers such as soluble interleukin-2 receptor (sIL-2R), chitotriosidase, KL-6, and neopterin have all shown promise in monitoring sarcoidosis activity and could be considered for integration into future versions of the score [27-29]. Additionally, quantitative PET parameters and BAL CD4/CD8 ratios, though not used here, may augment scoring in tertiary care centers with access to advanced diagnostics [30].

Our study has several notable strengths. It is grounded in real-world clinical data and follows a pragmatic design tailored to TB-endemic regions, where the diagnostic overlap between sarcoidosis and TB is a frequent clinical challenge. The use of robust statistical modeling, coupled with internal validation through bootstrap resampling, enhances confidence in the reproducibility and reliability of the YSDS.

However, important limitations must be acknowledged. The retrospective design inherently limits the ability to establish causality and may be prone to information bias due to variability in documentation. Additionally, the study was conducted at a single tertiary care center, which may not reflect the broader patient population seen in primary or secondary care settings. This single-center setting may limit generalizability, as the institutional referral patterns and availability of diagnostic resources could influence case selection. Moreover, selection bias is a possibility, as patients presenting to a tertiary care facility are more likely to have atypical or complex disease, potentially skewing the diagnostic spectrum. External validation through a prospective, multicentric study is therefore necessary to assess the applicability and diagnostic accuracy of the YSDS across diverse healthcare settings and populations.

 Thus, YSDS is an evidence-based, clinically grounded, and user-friendly tool that supports the diagnosis of sarcoidosis in complex clinical scenarios. It bridges a critical diagnostic gap in TB-endemic regions and shows promise for broader applications, including education, digital health, and outcome prediction.

## Conclusions

The YSDS is a novel, evidence-based tool developed from real-world clinical data to address the diagnostic ambiguity surrounding sarcoidosis in TB-endemic regions. By incorporating key radiologic, laboratory, histopathologic, and clinical features, it enables a more objective and accurate diagnosis. With high sensitivity and specificity, this scoring system could serve as a valuable clinical decision-making aid, especially in resource-constrained settings. External validation and prospective application are the necessary next steps for broader implementation.
